# Determinants of Mental Health in Europe

**DOI:** 10.1192/j.eurpsy.2025.97

**Published:** 2025-08-26

**Authors:** J. D. Lopez Moriñigo

**Affiliations:** Lundbeck España, S.A., Barcelona, Spain

## Abstract

**Abstract:**

Introduction This presentation will provide an up-to-date cross-national comparison of the individual, environmental and socioeconomic determinants of the European population mental health (MH). Methods The European House & Ambrosetti-funded 2023 Headway Mental Health Index 3.0 initiative collected data on 19 Key Performance Indicators (KPIs) in individual (e.g., smoking), environmental (e.g., air pollution) and socioeconomic (e.g., poor housing conditions) determinants of MH for the European Union 27 countries and the UK. KPIs scores were standardised in a 1-10 Likert Scale (1: worst performance; 10: best performance), thus allowing between-country comparisons of the relative performance. Unadjusted bivariate correlations between KPIs scores were run. Results Finland (8.0), Sweden and Estonia (7.5) had the lowest MH risk, while France (3.1) and Romania (2.8) had the highest MH risk. Smoking (r=-0.43, p=.021), alcohol use (r=0.57, p=.002), daylight hours (r=0.74, p<.001), ecoanxiety (r=-0.51, p=.005), air pollution (r=-0.46, p=.015), commuting time (r=0.42, p=.026) and Fragile State Index (r=-0.44, p=.018) correlated with overall MH status, thus emerging as common determinants of MH across the board. Conclusions The determinants of MH varied across European countries, although the correlation between determinants-based MH risk and MH status was relatively weak, including **‘**low-risk, poor MH status’ and **‘**high-risk, good MH status’ countries. Further non-tested determinants of MH and/or between-country differences in their responsiveness to the population MH needs may explain this discrepancy. These results may inform future evidence-based public MH policymaking and universal preventive strategies in Europe.

**Disclosure of Interest:**

None Declared

